# Objective Detection of Auditory Steady-State Responses (ASSRs) Based on Mutual Information: Receiver Operating Characteristics and Performance Across Modulation Rates and Levels †

**DOI:** 10.3390/audiolres15030060

**Published:** 2025-05-15

**Authors:** Gavin M. Bidelman, Claire McElwain Horn

**Affiliations:** 1 Department of Speech, Language and Hearing Sciences, Indiana University, Bloomington, IN 47408, USA; 2 Program in Neuroscience, Indiana University, Bloomington, IN 47405, USA; 3 Cognitive Science Program, Indiana University, Bloomington, IN 47405, USA; 4 School of Communication Sciences & Disorders, University of Memphis, Memphis, TN 38152, USA

**Keywords:** auditory evoked potentials (AEPs), auditory steady-state response (ASSR), evoked potential classification, F-test, objective audiometry

## Abstract

**Background**: Auditory steady-state responses (ASSRs) are sustained potentials used to assess the physiological integrity of the auditory pathway and objectively estimate hearing thresholds. ASSRs are typically analyzed using statistical procedures to remove the subjective bias of human operators. Knowing when to terminate signal averaging in ASSR testing is critical for making efficient clinical decisions and obtaining high-quality data in empirical research. Here, we report on stimulus-specific (frequency, level) properties and operating ranges of a novel ASSR detection metric based on mutual information (MI). **Methods**: ASSRs were measured in *n* = 10 normal-hearing listeners exposed to various stimuli varying in modulation rate (40, 80 Hz) and level (80–20 dB SPL). **Results**: MI-based classifiers applied to ASSR recordings showed that the accuracy of ASSR detection ranged from ~75 to 99% and was better for 40 compared to 80 Hz responses and for higher compared to lower stimulus levels. Receiver operating characteristics (ROCs) were used to establish normative ranges for MI for reliable ASSR detection across levels and rates (MI = 0.9–1.6). Relative to current statistics for ASSR identification (F-test), MI was a more efficient metric for determining the stopping criterion for signal averaging. **Conclusions**: Our results confirm that MI can be applied across a broad range of ASSR stimuli and might offer improvements to conventional objective techniques for ASSR detection.

## 1. Introduction

Auditory steady-state responses (ASSRs) are sustained evoked potentials typically elicited by amplitude or frequency modulated signals. ASSRs offer a rapid physiological assessment of hearing function and can be used to estimate full audiogram thresholds simultaneously in both ears [1,2,3]. ASSRs are preferred over other electrophysiological measures (e.g., auditory brainstem response, ABR) because response detection is based on a statistical comparison between signal and noise power in the evoked potential average rather than human waveform inspection [1,4,5,6]. This objectivity is beneficial as it avoids subjective operator interpretation and bias in determining the presence/absence of a response and the quality of the auditory evoked potential (AEP) recording [6,7,8].

Current approaches to analyzing ASSRs typically involve frequency domain measures where a statistic is applied to the response spectrum in order to determine the significance of the signal’s amplitude relative to the surrounding noise floor [1,4,5,6]. Several statistics have been proposed in the literature including the *F*-test, magnitude-squared coherence (MSC) [4,9], circular *T*^2^ statistic [10], *Fcric* [11], or *Fmp* statistic implemented in the Interacoustics-Eclipse system (https://tinyurl.com/28r8s4ry; Accessed 25 February 2025). In all cases, these statistics become more powerful with an increasing number of trials. As such, a stopping rule can be applied when a criterion value or significance level is achieved (e.g., *p* < 0.05). Such metrics are currently available in several commercial AEP systems. However, it remains unclear if these are the most optimal statistics for characterizing sustained AEPs. Arguably, metrics like the *F*-test are somewhat limited because they are usable only on specific features of the stimulus (e.g., power at the modulation frequency, *f_m_*). Consequently, these metrics cannot be broadly applied to sustained AEPs elicited by more complex sounds (e.g., multi-frequency, time-varying stimuli) that have proven more useful in characterizing central disorders of the auditory nervous system [2,12,13,14,15]. Novel statistical approaches might offer higher sensitivity and/or flexibility for detecting ASSRs and other sustained AEPs.

To this end, we began developing a novel statistical method for detecting sustained auditory potentials based on mutual information (MI) [8], a metric adopted from information theory and image processing (for review, see (for review, see [16]). The aim of our approach is to compare the spectrographic representations of the stimulus signal to that of the neural response [8]. While promising, our previous investigations were only proof of concept, testing MI using only a single suprathreshold stimulus. Thus, it remains unclear if MI can be more broadly applied to detect ASSRs elicited under a range of stimulus parameters including different modulation rates and levels. Furthermore, the criterion threshold for MI we used previously was estimated via computational modeling [8]. Thus, it is not clear from our previous studies whether this is the most appropriate criterion for detecting ASSRs evoked by different stimulus levels and rates or if it was idiosyncratic to the one stimulus in our prior report. New normative data reported here allowed us to address these open questions and characterize ranges for the MI metric based on its performance (e.g., sensitivity/specificity) in detecting a wider variety of ASSR responses. Understanding the performance of MI detection across different stimulus settings is critical if the response is to be eventually used for objective audiometry [1,2,3].

The present study aimed to more fully characterize the performance of an MI-based classifier for detecting ASSRs across a broader range of stimulus parameters. We assessed ASSR detection for responses recorded at different modulation frequencies (40 Hz, 80 Hz) to assess the metric’s dependence on stimulus modulation rate (and thus the putative site of the ASSR generator) (e.g., cortex vs. brainstem: [17]). Additionally, we parametrically varied the stimulus level across a large dynamic range (80–20 dB SPL) to evaluate the level dependence of MI in detecting ASSRs. This latter manipulation is important given the application of ASSRs for threshold estimation and objective audiometry [5,18]. Receiver operating characteristics (ROCs) allowed us to characterize how different choices of MI criterion values affect ASSR detection and thus allowed us to establish a normative operating range for the metric and guide its future implementation. Lastly, we evaluated the efficacy of the MI algorithm by comparing its application as a stopping criterion for signal ongoing averaging against other “gold-standard” statistical approaches (i.e., F-test) [1].

## 2. Materials and Methods

### 2.1. Participants

Ten young, normal-hearing listeners (5 male, 5 female; age: 23.7 ± 1.94 years) participated in the experiment This sample size is consistent with the sample sizes of other recent ASSR signal detection studies on normal-hearing listeners [11,19]. However, it is important to note that for the development of a statistical classifier which aims to segregate signal from noise, it is the unique number of datapoints that is most critical. In this regard, the aggregate data we use for statistical analysis and characterizing the parameter space of MI (e.g., see Figure 2) included a substantial (100,000 = 10 subjects × 4 levels × 2500 sweeps) number of unique data epochs. All participants had normal hearing thresholds (≤15 dB HL, octave frequencies 250–8000 Hz) bilaterally, were right handed [20], and were native speakers of American English. Participants gave written informed consent in compliance with a protocol approved by the University of Memphis Institutional Review Board (Protocol #2370).

### 2.2. Stimuli

ASSRs were evoked by sinusoidal amplitude-modulated (SAM) tones with a carrier frequency (*f_c_*) of 1000 Hz and modulation frequencies (*f_m_*) of 40 Hz or 80 Hz (100% modulation depth). Stimulus duration was 200 ms (including 5 ms onset/offset ramping to minimize onset components) following our previous report [8,21]. Stimuli were delivered binaurally via ER-2 insert earphones (Etymotic Research) at levels of 80, 60, 40, and 20 dB SPL using alternating polarity. In addition to these stimulus conditions, sham recordings were obtained by presenting stimuli with the inserts removed from participants’ ears [8,22]. Shams provided baseline, control recordings of “neural noise” [8,21].

### 2.3. Electrophysiological Recordings

ASSR recording procedures and stimuli were similar to those in our previous report [21]. EEGs were recorded between Ag/AgCl disc electrodes placed on the scalp at the high forehead by the hairline, referenced to linked mastoids (A1/A2) (mid-forehead = ground). Interelectrode impedances were ≤5 kΩ. Continuous EEG signals were digitized at 10 kHz (SynAmps RT amplifiers; Compumedics Neuroscan). EEGs were windowed [0–200 ms], filtered (30–1000 Hz), and averaged in the time domain to obtain ASSR waveforms for each stimulus. Listeners heard 2500 repetitions of the stimulus token presented at an interstimulus interval of 5 ms. Post-processing and analyses were performed using custom routines coded in MATLAB^®^ (v2024a; The MathWorks, Inc., Natick, MA, USA).

### 2.4. Mutual Information (MI) Detection Metric

We computed the mutual information (MI) between spectrographic representations of the stimulus and the neural response to index the degree to which neural responses captured spectrotemporal details of the acoustic input. Details of this metric are fully elaborated in our previous study’s bench testing MI for AEP detection [8,21]. MI is a dimensionless quantity (measured in bits) which measures the degree of linear and nonlinear dependence between two signals (A and B). In our specific case where A and B are two spectrograms, MI computes the dependence or similarity between the two images [16]. Effectively, the resulting value describes the similarity between the ASSR and stimulus spectrograms. We use this value to decide, statistically speaking, if the ASSR response is present or absent in the EEG recording.

MI was computed between the stimulus and each neural response spectrogram, allowing us to assess the degree to which neural responses reflect spectrotemporal properties of the evoking stimulus [8]. Spectrograms were computed using the “spectrogram” routine in MATLAB and converted to grayscale images. This routine computed a 2^14^ point FFT in consecutive 50 ms segments (Hamming windowed) computed every 3 ms [8] (Window length changes the spectral resolution of the resulting spectrogram, which could impact the computation of MI when comparing the stimulus and response spectrograms. In initial analyses, we varied the sliding window length parametrically from 25 ms to 100 ms. However, in pilot testing, we found no appreciable changes in the accuracy of response detection for different window lengths. Consequently, we adopted a 50 ms window, equivalent to a spectral resolution of 20 Hz. This is appropriate for resolving both 40 Hz and 80 Hz components and is consistent with our previous studies [8,15].). Time waveforms were zero-padded to minimize edge effects and ensure that spectrograms ran to the end of the signal’s duration. Identical parameters were used to compute both the stimulus and response spectrograms. SAM tone stimulus spectrograms were squared prior to computing MI to account for the half-wave rectification that is applied during the cochlear transduction process [21,23,24].

### 2.5. Receiver Operating Characteristics (ROCs) for the MI Classifier

After determining a criterion (i.e., decision rule) for MI statistically from our data (see the Results Section), we then applied this threshold (MI_θ_) as a binary classifier to ASSR and sham recordings. Recordings yielding MI ≥ MI_θ_ were classified as neural responses, whereas recordings with MI < MI_θ_ were considered noise (i.e., no response) [8]. Classifier performance was evaluated by computing standard signal detection theory and ROC metrics including true and false positive rates. ROC analyses also allowed us to determine the acceptable range of MI values that yielded above-chance detection of ASSRs from noise. For a given value of MI, sensitivity was computed as the percentage of actual ASSR recordings correctly identified; false positive rate was computed as the percentage of sham recordings (i.e., “neural noise”) erroneously classified as a biological ASSR response. ROC curves were constructed for each modulation rate (40 Hz, 80 Hz) and level (80–20 dB SPL) to characterize the overall performance of the MI classifier across the different stimulus settings.

### 2.6. Comparison of MI to F-Test

To test the efficiency of MI as a stopping criterion for signal averaging, we computed MI on a sweep-by-sweep basis as accumulating trials were added to the ongoing ASSR average. This was repeated separately for each level and modulation rate. Similarly, we compared the “online” development of MI against the well-known F-test [1,4] used in commercial ASSR recording systems (e.g., Bio-logic MASTER II; Intelligent Hearing Systems SmartEP-ASSR). While other detection metrics are available (e.g., MSC), we have previously shown that MI is most comparable in detection performance to the F-test (MSC performs more poorly) [21] and thus represents a stringent benchmark comparison. The underlying assumption of the F-test approach is that in the spectral domain, ASSR energy should be localized to a frequency bin near the stimulus modulation frequency; activity in adjacent bins contains only random noise, with zero mean and variance distributed equally across the noise bins [1]. The ratio of signal power to the sum of the powers in N adjacent frequency bins is distributed according to an F distribution with 2 and 2N-1 degrees of freedom [1]. In the current study, we used N = 12 frequency bins surrounding the target signal. We then compared our measured F-ratio against the critical F-value with 2 and 23 degrees of freedom and obtained a corresponding *p*-value for response detection. Traces yielding *p* < 0.05 were deemed to have response energy at the *fm* frequency that was significantly above the surrounding noise floor. Comparison between the MI and F-test statistical metrics allowed us to relate their performance and determine differences in their stopping rule for signal averaging, i.e., the number of trials where each measure detected the presence of ASSRs.

## 3. Results

### 3.1. ASSR Responses

**Figure 1:**
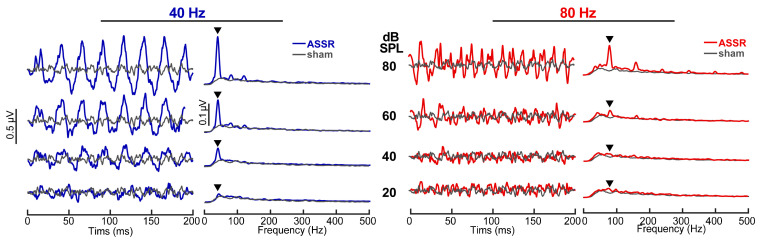
Auditory steady-state response (ASSR) waveforms and spectra elicited by 40 Hz (**left**) and 80 Hz (**right**) SAM tones (*fc* = 1 kHz) showing the level dependence of responses. ASSR waveforms show phase locking at the stimulus modulation rate and first few harmonics which progressively weakens with decreasing level, approaching the noise floor at ~20 dB SPL. Gray traces, sham recording in which the earphone was removed from the ear canal (i.e., EEG noise floor). ▼ = response energy at the *fm*.

ASSR time waveforms and spectra are shown for actual and sham recordings in Figure 1. The spectra illustrate response energy at the modulation rates (40 Hz or 80 Hz) and their upper harmonics for ASSR but not sham recordings (gray trace). These findings confirm that ASSRs contained robust phase-locked neural activity, whereas sham recordings contained no ASSR response (nor stimulus artifact) and are thus suitable for use as “catch trials” in testing our MI detection metric [21]. As expected, ASSR amplitudes also decreased with decreasing stimulus level and were only weakly above the noise floor at 20 dB SPL.

### 3.2. Performance and ROC Characteristics of MI Classifier

**Figure 2:**
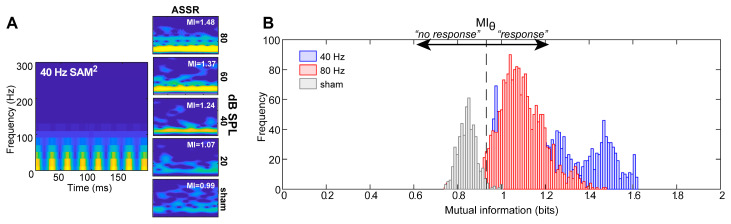
Characterizing the quality of ASSR recordings using mutual information (MI). (**A**) (*left*) A rectified stimulus spectrogram for a 40 Hz SAM tone stimulus. (*right*) Spectrograms of ASSRs recorded for a descending-level series. The inset of each panel indicates the MI computed between each response spectrogram and that of the stimulus [8,21]. With decreasing level, time–frequency representations of the neural ASSRs show less correspondence with those of the stimulus, as indicated by decreasing values of MI. (**B**) Signal detection theory analysis to determine an optimal criterion (MI*_θ_*) for the MI response classifier. Shown here is the distribution (probability density functions) of MI values for 40 and 80 Hz ASSRs (pooled across stimulus levels) and sham recordings. MI is always larger for true vs. sham recordings. The criterion MI*_θ_* = 0.93 segregates 95% of suprathreshold ASSRs from sham noise. From a classifier perspective, recordings containing MI > MI*_θ_* (=0.93) are predicted to contain a true neural ASSR response, whereas recordings with MI < MI*_θ_* are considered noise (no response).

Examples of MI computed between the 40 Hz SAM stimulus and responses are shown for different stimulus levels in Figure 2A. MI decreases at lower stimulus levels, indicating weaker dependence between the stimulus and ASSR response. At high intensities (80 dB SPL), ASSR spectrograms show strong dependence on the evoking stimulus spectrogram and MI is large. Nearer the threshold (20 dB SPL), ASSRs are dominated by background EEG noise, implying that the averaged neural response shares less information with the stimulus, which consequently yields a low MI (Note that non-zero MI is observed even for sham recordings, suggesting some shared time–frequency information between the stimulus and neural noise. We attribute this to myogenic noise of the EEG which is strong for frequencies <40 Hz. The SAM tone stimulus also has significant low-frequency energy below <40 Hz. Thus, even in the absence of a stimulus, spectral energy below <40 Hz in both the stimulus and “neural noise” can produce non-zero MI. This can be taken as the floor of the metric.).

Our first aim was to statistically determine a decision rule for MI for use in detecting ASSR responses. To this end, signal detection theory was used to determine an optimal criterion (MI*_θ_*) for the MI classifier from EEG recordings. Figure 2B shows the probability density functions of MI values for all trials and subjects for the 40 Hz and 80 Hz ASSR (pooling across levels) and sham recordings. On average, MI values range from ~1 to 1.5 across all stimulus combinations. All ASSRs were, to varying degrees, linearly separable along the MI decision axis compared to sham recordings, which elicited weak MI. In the current study, MI*_θ_* = 0.93 was taken as the criterion value because 95% of the data (i.e., MIs for ASSR responses) fell above this threshold; the false positive rate was 5%. From a signal detection standpoint, this implies that any arbitrary recording for which MI > MI*_θ_* will be predicted to contain a true ASSR response, whereas recordings with MI < MI*_θ_* are considered noise (no response). MI*_θ_* = 0.93 was determined to be the optimal decision rule for ASSR detection, though we show later that a range of values around MI = 1 are also sufficient.

**Figure 3:**
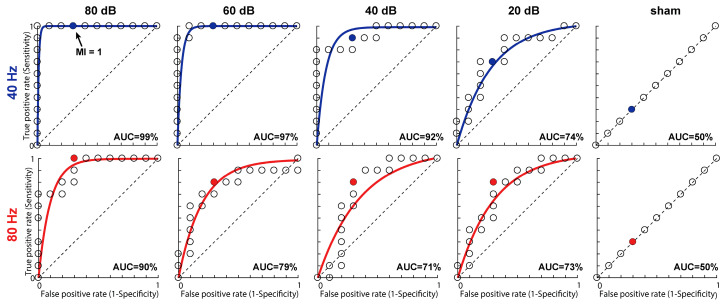
Receiver operating characteristic (ROC) curves for ASSRs evoked by different modulation rates and levels. ROC curves measure tradeoff in sensitivity and specificity with different MI cutoffs (marked by different points along each curve). Individual points denote the true positive (sensitivity) vs. false positive (1-specificity) rates for various values of MI in distinguishing true from sham recordings based on 2500 sweeps. Dotted lines correspond with chance performance (i.e., *d*′ = 0). With an optimal value of MI for signal–noise segregation (MI*_θ_* = 0.93; see Figure 2), classification accuracy (AUC) is 99% for 40 Hz ASSRs at 80 dB SPL and 90% for 80 Hz ASSRs. Classification accuracy is better for low (40 Hz) compared to high (80 Hz) modulation rates and high- vs. low-level stimuli.

Classifier performance of the MI metric is shown in Figure 3 as ROC curves. Each panel represents the true (sensitivity) vs. false positive (1-specificity) rate for distinguishing ASSRs from sham recordings at different stimulus levels. The bowing of the ROC curve toward the upper left corner is indicative of robust sensitivity in segregating signal from noise (i.e., higher *d*-prime). Each individual datapoint represents the true/false positive rate for a different choice of MI*_θ_*. A criterion located at the maximum curvature of the ROC curve represents the optimal decision rule for classification, one which produces the highest sensitivity while minimizing false positive detection (i.e., erroneously labeling a noise recording as an ASSR).

With decreasing levels, ASSRs become more difficult to segregate from EEG noise, as evident by the ROC curves approaching chance performance (dotted lines) at 20 dB SPL. Overall classification accuracy for the 40 Hz responses is near ceiling (99%) at 80 dB SPL, as indicated by the area under the curve (AUC) [25]. Classification accuracy weakens with decreasing level, indicating that discriminating ASSRs from noise is more difficult nearer to the threshold. Nevertheless, classification remains high (74%) for the 40 Hz responses at 20 dB SPL. Accuracy in detecting 80 Hz responses is 10–15% poorer compared to 40 Hz responses but remains well above chance (73%) even at the lowest intensity tested. These operating characteristics demonstrate that the MI between a stimulus and neural response provides an objective means for detecting ASSRs across various levels and modulation rates.

### 3.3. Acceptable Ranges of MI for ASSR Detection

**Figure 4:**
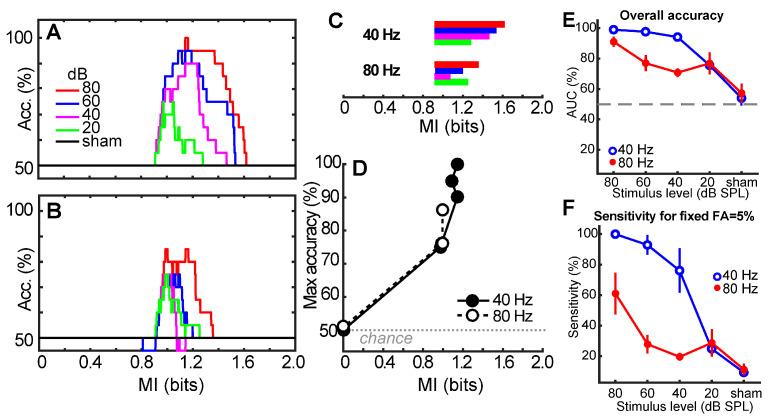
Acceptable MI values for detecting suprathreshold ASSRs across stimulus levels and modulation rates. (**A**,**B**) Level-dependent classification accuracy for the 40 Hz (**A**) and 80 Hz (**B**) responses. Each family of functions shows the overall accuracy in correctly detecting ASSRs from noise using different MI cutoffs. (**C**) Range of acceptable MI values for classifying 40 and 80 Hz ASSRs above chance levels. Ranges were extracted from the width of each level-dependent accuracy function shown in panels A and B. Color scheme, same as in panel A. (**D**) Max accuracy for detecting ASSRs and the corresponding MI. Max accuracy was extracted from the peak of each level-dependent accuracy function of panel A and B. Note that some points for the 80 Hz responses overlap. (**E**) Overall accuracy across stimulus levels and modulation rates. Accuracies were extracted from ROC functions (e.g., Figure 3) as the area under the curve (AUC). (**F**) Sensitivity of the MI metric for a fixed false positive rate of 5%. Response detection accuracy and sensitivity are better for 40 Hz compared to 80 Hz responses, decrease with decreasing stimulus level, but remain well above chance. Error bars = −95% CI.

While the statistically derived criterion MI_θ_ = 0.93 represents the optimal threshold for detecting ASSRs (5% false positive), our ROC characterizations reveal that there is a range of acceptable MI values around a convenient MI ≈ 1 that could be used to reliably detect neural responses. Figure 4 shows the overall accuracy of detecting ASSRs from shams for different choices of MI for 40 Hz (Figure 4A) and 80 Hz (Figure 4B) responses. Each family of functions shows the overall accuracy in correctly distinguishing ASSRs from noise using different MI cutoffs. The reduction in peak accuracy across curves indicates a level-dependent effect in classification accuracy. Consistent with ROC results, MI is less robust at detecting ASSRs evoked by weaker stimulus levels. Nevertheless, there is a range of MI values that allow for above-chance detection of the response (Figure 4C).

MI ranges were extracted from the width of each level-dependent accuracy function shown in panels A and B and show the acceptable range of MI cutoff thresholds that allow for above-chance detection (Figure 4C). For the 40 Hz response, acceptable values of MI range from 0.9 to 1.6 for high-level (80 dB) stimuli. This allowable range is reduced with decreasing level; 40 Hz responses are detectable at 20 dB with MI values between 0.9 and 1.3. Similar results were obtained for the 80 Hz responses, although the acceptable MI range was reduced at both high- (0.9–1.4) and low-level (0.9–1.2) stimuli.

The corresponding 95% confidence intervals for MI across levels were similar for 40 Hz ([0.99 1.14]) and 80 Hz responses ([0.99 1.13]). Notably, both 95% CIs included 1.0, suggesting that a simple value of MI = 1 could be applied to ASSRs for response detection. MI = 1 is also convenient in terms of its information–theoretic interpretation: MI = 1 means that ASSR detection is achieved when “1 bit” of information is transferred (is shared) between the stimulus and EEG response spectrograms.

MI values corresponding to maximum classification accuracy (i.e., peak of functions in Figure 4A,B) are shown in Figure 4D. Maximum accuracy is obtained with MI ≈ 1 (cf. MI*_θ_*). Collectively, these results help provide a normative tolerance range and optimal choice of MI values for using it as an ASSR classifier.

Typically, the performance of a diagnostic or detection method is evaluated by considering the sensitivity and specificity of the measure. However, it is also useful to evaluate a diagnostic’s true positive rate (i.e., sensitivity) for a fixed false positive rate (e.g., 5%). Figure 4E,F show the overall accuracy of the MI metric (AUC) and sensitivity at a fixed 5% false positive rate (i.e., 95% specificity) across stimulus levels and modulation rates. For 40 Hz ASSRs, performance ranges from 100/95% sensitivity/specificity at 80 dB SPL to 20/95% sensitivity/specificity at 20 dB SPL. For 80 Hz ASSRs, performance ranges from 55/95% sensitivity/specificity at 80 dB SPL to 20/95% sensitivity/specificity at 20 dB SPL.

### 3.4. MI as a Criterion for Terminating Signal Averaging

**Figure 5:**
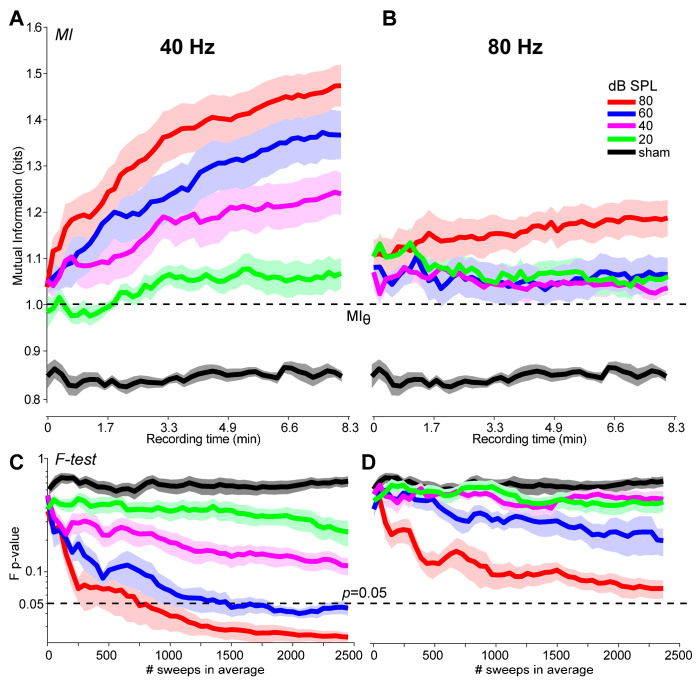
A comparison of the growth in MI with the *F*-test during online ASSR recording. Sweep-by-sweep ASSR detection based on MI for the 40 Hz (**A**) and 80 Hz (**B**) responses at each level. (**C**,**D**) Improvements in ASSR detection using the conventional *F*-test procedure [1]. Dotted lines denote the criterion threshold for ASSR detection under each metric (MI = 1.0; *F*-test: *p* < 0.05). The abscissa shows both the number of sweeps and corresponding recording time for online ASSR averaging. Shading = ±1 s.e.m.

In addition to detection, an objective metric should be suitable as a stopping criterion for online signal averaging. Figure 5 shows the growth in MI and the conventional *F*-test [1,4] metric during ASSR recordings as a function of the number of trials in the ongoing average. In this figure, we set the MI threshold to the convenient value of MI = 1, the approximate midpoint of the metric’s 95% CI (see Section 3.3). In general, each metric improves with additional trials and asymptotes as the running AEP stabilizes. Response growth is faster for 40 Hz relative to 80 Hz responses and at higher (80 dB SPL) compared to lower (20 dB SPL) intensities. For high-level 40 Hz ASSRs, responses exceed the MI and F-test stopping criteria (MI = 1.0; F-test: *p* = 0.05) by ~50 and 750 sweeps, respectively, corresponding to <1 min (MI) vs. 2.5 min (F-test) of recording time. More extended recording durations (sweeps) are needed for detecting low-level ASSRs and 80 Hz responses, which sometimes do not achieve the criterion thresholds (e.g., Figure 5D). As an expected control, MI remains invariant sweep to sweep for noise sham recordings.

**Figure 6:**
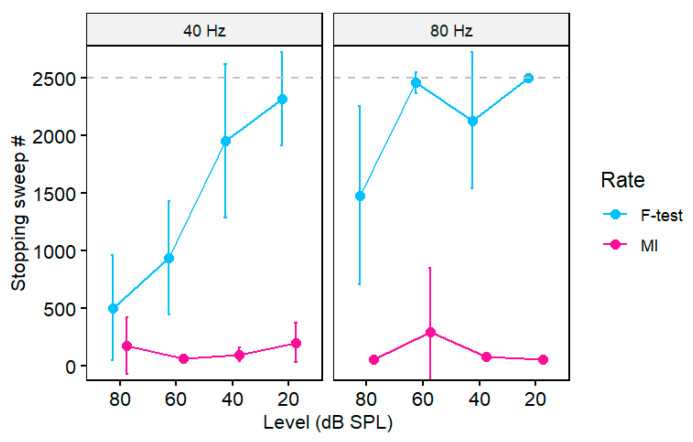
A comparison of the stopping time (# of sweeps) for ASSR recordings between the conventional F-test and MI (MI = 1). Longer recording times are needed for lower-level stimuli. MI generally outperforms the F-test with earlier response detection (i.e., faster recording times). Dotted line = max (ceiling) number of sweeps in the recording session. Error bars = −95% CI.

We conducted a mixed-model ANOVA (lme4 package in R [26]) on the stopping sweep number to assess differences in the recording time needed under each metric. The fixed effects were rate, level, and test type (subject served as a random effect). Not all recordings reached the stopping criteria, especially for low-level, 80 Hz stimuli (e.g., Figure 5D). These missing observations were coded as 2500, the maximum number of sweeps in our recording session. The ANOVA revealed a significant three-way rate x level x test interaction [*F*(3, 144) = 2.53, *p* = 0.059, $\eta_{p}^{2}$ = 0.05] (all lower-order two-way and one-way effects were also significant) (Figure 6). Tukey-corrected multiple contrasts revealed that the interaction was attributed to better performance of MI over the F-test in all but the 40 Hz, 80 dB SPL stimulus condition. These results indicate that MI outperformed the F-test across decreasing stimulus levels and modulation rates and detected the ASSR response in a fewer number of trials.

## 4. Discussion

There have been few developments in the ASSR literature over the past decade outside of industry manufacturers [27]. The majority of independent research efforts have focused on simultaneously recording the response with other evoked potentials (e.g., brainstem + cortical AEPs) [28,29,30,31], efficient protocol development and the use of the response as a diagnostic in certain clinical populations [32,33], or optimizing stimulus selection for enhancing the response [34]—the so-called “Next-generation” ASSR using chirp sounds [35,36,37].

Here, we report on the tolerance and operating range of an objective statistical approach to detect ASSRs based on mutual information (MI) [8]. The technique quantifies the quality of ASSRs by considering the linear and nonlinear dependences between the rich time–frequency information provided by the signal and response spectrograms. Our previous report’s bench testing of the MI classifier demonstrates its superiority over “gold-standard” (i.e., visual subjective) judgments of human observers [8] and other objective techniques for ASSR detection (e.g., MSC, *F*-test) [21]. Here, we extend these previous findings by showing that MI can be used for response detection across a broader range of ASSR-evoking stimuli including different combinations of levels and modulation rates that are used in the audiological clinic. Given that the MI tracks with level, it can be used to guide threshold searches. We also found that MI yields higher efficiency in detecting ASSRs in shorter recording times than conventional statistical algorithms [21]. The present results reaffirm the multiple benefits of MI and suggest that the statistic might be a useful tool to reduce valuable recording time during auditory electrophysical testing in the clinic.

We found that the overall performance accuracy in distinguishing true neurobiological responses from noise using our MI metric was >90% for high-level stimuli (80 dB SPL) and remained well above chance (73%) for levels nearer to auditory threshold (20 dB SPL). More importantly, our results establish a normative tolerance range for MI criterion values (MI = 0.9–1.6) that allow for robust detection of ASSRs across different modulation rates and intensities. However, as determined by ROC analyses, the most optimal classification of ASSRs is achieved with the criterion MI*_θ_* = 0.93. Though for the sake of convenience, MI = 1.0 would suffice in robustly identifying ASSRs from EEG noise (Figure 4), such that “1 bit” of information must be transferred between the stimulus and EEG response to confirm that an ASSR is present. Lastly, we showed that MI increases monotonically with an increasing number of stimulus presentations (i.e., trials) and can, for some stimulus conditions, detect ASSRs in a fewer number of trials compared to conventional ASSR detection procedures, i.e., the F-test (i.e., F-test; [1,4]).

The more comprehensive stimulus set used in this study compared to our previous reports [8,21] allowed for a more complete characterization of MI’s effectiveness as an ASSR classifier. Several observations are worth noting regarding the metric’s performance and limits. First, while MI can successfully detect the presence of ASSRs at different modulation rates (Figure 3), we found that overall accuracy was generally higher for 40 Hz compared to 80 Hz responses. The higher classification for 40 Hz compared to 80 Hz suggests that MI-based detection may be more sensitive to cortical rather than subcortical auditory phase-locked activity [17,38]. Critically, however, MI detection was still well above chance in all cases (Figure 4) and overall testing time was reduced compared to the F-test (Figure 6). The more optimal performance at 40 vs. 80 Hz is a common bias in the ASSR literature [39] and is likely due to the higher signal-to-noise ratios and more robust amplitudes of ASSRs with low vs. high-frequency modulation rates, as shown in the present study in Figure 2 [15,40,41]. Indeed, by early adolescence, the 40 Hz response is nearly twice the amplitude of the 80 Hz response [42]. Moreover, unlike their 80 Hz counterparts, 40 Hz responses are highly dependent on subject state: low *fm* responses are reliably recorded only in awake individuals [41,43,44] and are eradicated with anesthesia [38,40]. Despite this typical low-frequency bias in the ASSR, we found that the overall testing time was reduced using MI compared to the F-test (Figure 6). Our results therefore suggest that MI could offer a means to collect sustained ASSR/AEP data in a more time-optimized manner and reduce valuable recording time in the clinic (present study; [8,21]).

Secondly, we found that MI has a smaller useable range (Figure 4C) and lower accuracy/sensitivity (Figure 4D) for low-level, 80 Hz stimuli. This would limit the metric’s application for threshold testing [45], particularly in infants [46]. Additionally, neural generators of ASSRs are dependent on the frequency of the stimulus modulation rate; high frequencies (80 Hz) evoke brainstem generators, whereas low frequencies (40 Hz) recruit cortical sources [17,38]. Thus, the fact that we observe superior performance for 40 Hz stimuli across the board implies that MI might be more useful for monitoring cortical rather than subcortical neural activity.

Lastly, sweep-by-sweep tracking of MI confirmed the metric’s efficiency as a stopping rule for ASSR signal averaging. In this regard, we found that MI was able to detect 40 Hz ASSRs within ~1 min, corresponding to <50 stimulus trials. In contrast, using the F-test required considerably more stimulus presentations; ~750 sweeps (2.5 min of testing) were needed to detect the 40 Hz response at 80 dB SPL and ~1500 sweeps at 60 dB SPL. Moreover, our 80 Hz ASSRs never achieved the F-test criterion, indicating that more than 2500 trials would be needed to detect those responses. While our data show that MI can offer a more efficient stopping rule for terminating averaging compared to the *F*-test, from a practical standpoint, this improvement in testing time (1–2 min) is probably negligible. Nevertheless, our data indicate that under certain stimulus conditions, MI can detect ASSRs in half the number of trials (i.e., twice as efficient) as the gold-standard *F*-test.

More broadly, MI is an information–theoretic measure that is “distribution free” and therefore requires fewer assumptions than other statistical approaches (e.g., *F*-test) which utilize parametric (distribution-based) statistics. This is another benefit as auditory responses tend to be highly nonlinear. Thus, MI might offer an improvement upon older and other more recent ASSR detection statistics for use in objective audiometry [11,19]. However, unlike other metrics, MI can be easily applied to time-varying signals beyond the ASSR [8]. Thus, in addition to clinical application as a stopping criterion for ASSR audiometry and threshold testing, it can be readily applied to electrophysiological responses like the speech-evoked FFR [8]. This would be another major clinical benefit as FFRs have become popular in assessing complex auditory function including central auditory processing disorders [12], aging [47,48], cognitive decline [13], and learning-related plasticity [49,50].

## 5. Conclusions

The application of the MI metric to electrical response audiometry and ASSRs may provide clinicians and researchers with a more robust tool to objectively evaluate the presence and quality of sustained auditory AEPs, including fast testing time. The calculation of MI could be easily incorporated into commercially available AEP systems similar to other statistical detection metrics (e.g., *F-test*, *F_sp_*, *Fmp*, *MSC*) already available in clinical hardware (MASTER system, Interacoustic Eclipse, Intelligent Hearing Systems). Implementation would require relatively straightforward signal processing to first compute the time–frequency spectrograms of the (i) stimulus and (ii) response epoch followed by MI between i and ii. MI can then be tracked in a running manner during online averaging as trials are accumulated (e.g., as in Figure 5). Signal averaging could then be terminated immediately once the criterion value (established here: M ≈ 1) is exceeded, saving valuable clinic time and guess work. Still, future studies are warranted to assess the performance of MI in clinical populations (e.g., infants, hearing loss) where testing times can be much longer than in listeners with normal hearing [51].

## Data Availability

The data presented in this study are only available on request from the corresponding author due to privacy reasons.
